# The Role of the Mirror System in Influencing Musicians’ Evaluation of Musical Creativity: A tDCS Study

**DOI:** 10.3389/fnins.2021.624653

**Published:** 2021-04-08

**Authors:** Barbara Colombo, Reid Anctil, Stefania Balzarotti, Federica Biassoni, Alessandro Antonietti

**Affiliations:** ^1^Neuroscience Research Lab, Champlain College, Burlington, VT, United States; ^2^Department of Psychology, Catholic University of the Sacred Heart, Milano, Italy

**Keywords:** mirror system, auditory mirror system, creativity, musicians, empathy, tDCS, left ventral premotor cortex, emotions

## Abstract

Evidence reported in the literature suggests that the mirror system not only plays a role in recognizing motor action but also fosters a better understanding of other people because it helps an individual assume another’s perspective. This led to the idea, supported by research findings, that people with higher empathy scores should show higher activation of the mirror system. Recently, it has been hypothesized that a purely auditory mirror system exists. In this study, we aimed to explore the possibility that this system might play a particular role for musicians. Specifically, this system would impact their response to a new piece of music by using non-invasive brain stimulation to modulate the activation of the mirror system. A sample of 40 young musicians was involved in this study. Half of the participants were randomly assigned to a cathodal stimulation condition, while the other half was used as a control. After listening to a new piece of music, participants were asked to rate the creativity of the piece (by focusing on how interesting, innovative, and exciting the piece was) as well as their general emotional response to it. Their empathy levels were also assessed using the Interpersonal Reactivity Index (IRI). Results showed that the cathodal stimulation of the mirror system negatively affected both the perception of creativity (level of innovation) and the emotional response to the music. There was no significant difference in the ratings of how interesting the piece was perceived. The effect was mediated by the individuals’ level of empathy. Specifically, empathic concern and fantasy dimensions increased the evaluation of creativity. Results also showed that participants reported less emotion with a negative valence in the cathodal stimulation condition.

## Introduction

### Theoretical Background on the Mirror Neuron System

The mirror neuron system (MNS) has had a large impact on the psychological community since the discovery of mirror neurons in the ventral premotor (F5) area of Macaque monkeys in 1992. Mirror neurons have become widely investigated due to their unique nature (Cattaneo and Rizzolatti, 2009; Kilner and Lemon, 2013; Jeon and Lee, 2018). Mirror neurons diverge from motor and sensory neurons due to the fact that they become active both with the performance of an action and with the observation of another performing the action (Rizzolatti, 2005; Kilner and Lemon, 2013).

Since the MNS was discovered, research on the MNS has branched out from studying the response to simple motor actions. An interesting discovery is the fact that the auditory system is also involved. To be more specific, a group of audiovisual neurons in the ventral premotor F5 area seems to be able to discriminate between different actions with about 90% accuracy when only seen or only heard (Kohler et al., 2002; Keysers et al., 2003). Further research on humans supports the fact that there are auditory mirror neurons that activate in response to the sounds of actions we are capable of performing (Gazzola et al., 2006). This makes sense when reflecting on the fact that the representation of sensory and motor information in the brain seems to be integrated at many levels: For this reason, seeing or hearing action-related stimuli may automatically cue the movements required to respond to or produce them, in order to guide perception of musical stimuli (Stephan et al., 2018). This line of research has led to interesting discoveries, including the role that the MNS might play, in humans, in facilitating or mediating the understanding of music (Jiang et al., 2019).

Focusing more specifically on response to music in musicians, Lahav et al. (2007) found that auditory mirror activation only occurred when listening to a passage from a song that participants were taught to play on the piano. This did not occur when listening to a passage of an unfamiliar song. This suggests that only sounds within our motor repertoire will activate the auditory MNS. The researchers speculated that participants may not have responded to songs they had not been taught, due to their unfamiliarity with the instrument and music in general (Lahav et al., 2007). A similar study by Bangert et al. (2006) suggested that professional musicians have a greater understanding of the motor and auditory parts of piano playing, allowing them to still have significant understanding of the piano without either motor or auditory stimuli and supporting the idea that professional musicians would show more MNS activity with new music than would non-professionals. More data also support the fact that mirror neuron activation is modulated by musical expertise and that MNS activation in musicians may stem from imagining themselves playing the piece, so it is most likely stronger when they listen to music performed on their main instrument (Hou et al., 2017).

Evidence from recent studies that focus on the MNS (Ramachandra et al., 2009) suggests that the MNS may serve as a common neural substrate for processing not only motor information but also emotional and other higher-level cognitive information. Researchers (Warren et al., 2006; Banissy et al., 2010) explored the possible role of the auditory MNS in engaging different emotional systems as well as helping to discriminate auditory emotions, highlighting how distinct functional subsystems within the auditory–motor mirror network respond preferentially to emotional valence and arousal properties of heard vocalizations. To be more specific, Warren et al. (2006) reported that listening to non-verbal vocalizations can trigger an automatic preparation of responsive gestures, an effect that is greatest for positive-valence and high-arousal emotions. Yet the specific role played by this system when professional musicians listen to music where their main instrument is played has not been explored yet, to our knowledge.

### The Present Study: The Relationship Among Creativity, Empathy, and the MNS

The present study aimed to explore, at a preliminary level, if and how altering the activation of the MNS affects either the emotion response to music or the evaluation of musical creativity by professional musicians.

The added focus on creativity is derived from two lines of research. The first one highlights the relationships between creativity and empathy (and hence creativity and the MNS) and the second one the relationships among creativity, music, and empathy. The first relationship starts from the idea that creativity is linked to and supported by social aspects (Glaveanu et al., 2013), implying that a creative person will benefit from being connected to other people’s minds and feelings (Form and Kaernbach, 2018), aspects that are also promoted by the activation of the MNS. This view is supported by the empirical evidence showing that creative activity can be used as a tool to directly promote empathy and indirectly promote other social–emotional skills (Morizio, 2021). For example, painting in a virtual-reality environment has been reported to promote both creativity and empathy (Gerry, 2017), and creative dance has been used effectively to enhance the link between creativity, social interaction, and the MNS (Batson, 2013). The second line of research, starting from the idea that music can be seen as a specific type of creative thinking (Antonietti and Colombo, 2014), suggests that empathy influences the appreciation of performing and creative arts, including music (Wöllner, 2012). This could be linked to the fact that the performance of music, as it is true of creative activities, as discussed above, is claimed to be a social activity, and hence, even just listening to music has been shown to involve empathy (Cross et al., 2012; Wöllner, 2012; Balteş and Miu, 2014; Sittler et al., 2019). To be more specific, Kawakami and Katahira (2015) reported that fantasy and perspective taking, two sub-components of trait empathy assessed by the Interpersonal Reactivity Index (IRI) questionnaire, are correlated with the emotional response to sad music. Following this line of reasoning, empathy emerges as a variable that impacts the mirror system, as also discussed in a recent meta-analysis (Bekkali et al., 2020). Research highlights that individuals who showed high motor and facial mimicry more frequently had higher empathy scores (Sonnby-Borgström et al., 2003). Moreover, neuroimaging studies show positive correlations between the activation of the MNS and empathy scores from the IRI (Baird et al., 2011). These studies included both auditory and visual paradigms, suggesting that empathy could possibly play a part in both functions of the MNS. However, Baird et al. (2011) speculated that the brain regions associated with empathy could be related to partially different brain networks, depending on the specific form of empathy investigated (e.g., motor, emotional, and cognitive empathy), and hence, more studies that focus more specifically on specific forms of empathy are needed. They also explain that mirror neurons account for only a minority of cells in the brain regions associated with the MNS but that activation in the corresponding areas in humans has been heavily attributed to mirror neurons, prompting further clarification and study (Baird et al., 2011). Possible involvement of the MNS was also reported by Schnell et al. (2011), who found that cognitive empathy involves references to an individual’s own affective state. Our own affective state impacts how we understand the affective state of others. This way of referencing ourselves to understand others is similar to how we reference our own motor repertoire in order to understand the sound produced by another (Gazzola et al., 2006; Lahav et al., 2007). Gazzola et al. (2006) showed that participants who scored higher on a perspective-taking empathy scale showed stronger activation of the mirror system with data supporting that it was not due to lack of attention. This evidence suggests that empathy could play a role in the MNS but is not enough to declare a definitive relationship.

### Aims and Hypotheses of the Study

Starting from these premises, in this study we investigated if the auditory MNS might act in a specific way upon professional musicians. To do so, we used transcranial direct current stimulation (tDCS) to inhibit the activation of the MNS. Based on the research discussed above, we investigated how professional musicians would respond (both emotionally and cognitively) to a new piece of music involving the instrument they play. To be more specific, we investigated how cathodal tDCS stimulation of a musicians’ brain area associated with the MNS would affect their judgment of how creative the music was as well as their emotional response to it. Empathy has also been shown to have a relationship with the MNS, although the exact nature of this relationship has not been established yet (Gazzola et al., 2006; Baird et al., 2011; Schnell et al., 2011). Therefore, it was added as a covariate using the IRI (which has been used in the research mentioned above that focused on the relationship between music and empathy) in order to identify any possible moderating effects.

We expected that participants who received cathodal tDCS would rate the music as less creative when compared to participants in the sham condition, given the fact that their auditory MNS would be impaired. We also expected cathodal tDCS to impact self-reported emotional reaction to music, by way of reducing the intensity of reported emotions. This hypothesis is linked to the literature discussed above, which highlights the possible role of the MNS in processing emotional information. Since this specific processing seems (as noted above) linked specifically to the emotional valence and arousal/control associated with specific pieces of music, in this study we decided to assess these aspects by using the Geneva Emotion Wheel (GEW) (see details below), which focuses on assessing individual emotional responses on these two axes. Finally, given the relationships between creativity, empathy, and music, we expected individual levels of empathy to be positively associated with creativity ratings and to play the role of significant mediators between our two main variables (tDCS and evaluation of creativity).

## Materials and Methods

The study has been reviewed and approved by the Champlain College Institutional Review Board (IRB) (IRB protocol number: IRB000143).

### Sample

Forty young musicians (age range: 18–22, mean = 19.80; SD = 1.56; *F* = 15) joined the study and were randomly assigned either to the experimental group (cathodal stimulation) or to the control group (sham stimulation).

Participants were screened before being invited to join the experiment by checking that their principal instrument would be either the piano, violin, or cello (the instruments played in the piece of music used during our experiment; see below for details). We also verified that they would practice a minimum of 4 h/day and have performed in public in a professional setting at least five times. Of the recruited participants, 16 were pianists, 14 were violinists, and 10 were cellists.

### Apparatus

#### tDCS Equipment

In this study, we used 1300A 1 × 1 Transcranial Direct Current Low-Intensity Stimulator by Soterix Medical to deliver brain stimulation to our participants. We used two 5 × 5 cm rubber electrodes enveloped in saline-soaked sponges covered with conductive gel. For the experimental conditions (cathodal), the stimulation was set at 1.5 mA (current density: 0.02857 mA/cm^2^) for 20 min. In the control (sham) condition, the equipment started the stimulation normally and ramped up to the target intensity of 1.5 mA; it decreased to 0 mA after 5 s. This gave participants the impression of actually receiving stimulation, when in reality the stimulation lasted only 5 s, thus having no actual effect on brain functions.

For the experimental condition, the electrodes were placed on the left ventral premotor cortex using the 10–20 system (F5 location). The anodal electrode was placed on the upper right forearm. The same montage was used for the sham condition.

#### GEW

The GEW (Scherer, 2005; Scherer et al., 2013) measures emotional reactions to objects, events, and situations. Participants were asked to indicate the emotion(s) they experienced by choosing intensities for a single emotion or a blend of several emotions out of 20 different options. The emotions are arranged in a wheel shape with the axes being defined by two major dimensions of emotional experience: high vs. low control and positive vs. negative valence. Five degrees of intensity are being proposed, represented by circles of different sizes. In addition, “None” (no emotion felt) and “Other” (different emotion felt) options are provided. The GEW has been used to assess affect and emotional responses in many different research designs, as critically discussed in a GEW rating study (Sacharin et al., 2012). Results from these studies support the validity of the GEW. Other studies reported that participants tend to prefer the GEW over alternative measures (Desmet et al., 2000) and to judge the GEW as clear to understand and useful in differentiating between emotions (Caicedo and Van Beuzekom, 2006). The GEW has also been used specifically to assess emotional responses to music in neuropsychological experiments (Dutta et al., 2020).

#### Creativity Evaluation

We asked participants to rate specific factors that have been reported in the literature to be associated with creativity: interest (Fürst and Grin, 2018; Li et al., 2018; Moreira et al., 2020), innovation (Acar et al., 2017; Rietzschel and Ritter, 2018; Lee et al., 2020), and excitement (Paulus and Nijstad, 2019; Fink et al., 2020). Participants were asked to rate the creativity of the musical piece by rating how interesting, innovative, and exiting the piece was on a 9-point Likert scale. To be more specific, participants were told: “You are now asked to evaluate the creativity of the piece you just listened to. How interesting/innovating/exciting you think it is?”

#### IRI

The IRI (Davis, 1980, 1983) is a multidimensional measure of dispositional empathy that is widely used to assess empathy and has a strong validity portfolio (Keaton, 2017). It is a self-report questionnaire, which includes 28 items answered on a 5-point Likert scale ranging from “Does not describe me well” to “Describes me very well.” It consists of four subscales: perspective taking (PT, the tendency to spontaneously adopt the psychological point of view of others in everyday life); empathic concern (EC, the tendency to experience feelings of sympathy and compassion for unfortunate others); personal distress (PD, the tendency to experience distress and discomfort in response to extreme distress in others); and fantasy (FS, the tendency to imaginatively transpose oneself into fictional situations). The relationships among subscales have been statistically tested by analyzing the validity of a hierarchical structure of the IRI (Pulos et al., 2004).

#### Music

*Dreaming Cities* is a five-movement piano trio (violin, cello, and piano) by Damon Ferrante. In this experiment, participants listened to the third movement. The third movement is a slow movement whose material is a variation of the musical theme that occurs at the beginning of the work. The third movement’s sparce, lyrical texture highlights the characteristic musical voices of each instrument. It was not written with a specific emotional tone in mind, but, rather, focusing on the slow, melodic interplay of the instruments. This piece of music was not familiar to any participant (a familiarity check was performed at the end of the experiment).

### Procedure

After reading and signing the consent form and before starting the experimental procedure, participants were asked by researchers for any questions they might have.

The consent form included information about the tDCS equipment and possible side effects, listed exclusion criteria [e.g., personal or family history of seizures, traumatic brain injury (TBI) in the previous year, pregnancy, or any metallic implants in the skull], described the experiment and the different tasks, and reminded the participants that they would be free to leave the experiment at any time and to ask for their data to be deleted. We also explained how we were going to guarantee participants’ confidentiality by only using anonymous codes to identify the records.

After placing the electrodes and starting the tDCS stimulation (either actual stimulation or sham) and waiting 60 s to be sure that the equipment was functioning properly and no side effects were reported, participants were instructed to close and relax for 5 min to wait for the tDCS to have an effect. After that, participants were asked to open their eyes and listen to the piece of music selected for our experiment.

When the music was over, participants were asked to fill out the GEW, the creativity evaluation, and the IRI.

After that, the electrodes were taken off. Participants were asked if they had any questions, debriefed, and thanked for their participation.

## Results

### Effects of Brain Stimulation on Emotional Response and Creative Evaluation

To explore the effects of the brain stimulation on emotional reaction as well as creative evaluation of the musical piece, we ran a general linear model (GLM) multivariate analysis of variance (MANOVA), using the condition as an independent variable and the three creative evaluation scales (interest, innovation, and excitement) and self-report of emotional response (categorized into two variables: sum of positive valence emotions and sum of negative valence emotions) as dependent variables. We added the IRI subscales as covariates to control for their effect.

Mean scores and standard deviations are reported in Table 1.

**TABLE 1:** Mean scores and standard deviation for self-report evaluation of creativity and emotional response.

Self-report	Condition	Mean	Standard deviation
Creativity—Interesting	sham	6.20	1.88
	cathodal	6.40	1.39
Creativity—Innovative	sham	5.20	2.42
	cathodal	4.90	2.22
Creativity—Exciting	sham	4.40	2.30
	cathodal	4.00	2.15
Emotions—Positive Valence	sham	31.05	10.32
	cathodal	31.95	9.98
Emotions—Negative Valence	sham	10.35	4.70
	cathodal	4.15	1.81

The test of between-subject effects returned a significant main effect of stimulation condition on the evaluation of the creativity of the piece. Two of the considered dimensions were significantly affected: how innovative the piece was (*F*_1,34_ = 45.76, *p* < 0.001, η^2^ = 0.57) and how exciting (*F*_1,34_ = 53.73, *p* < 0.001, η^2^ = 0.61). In both cases, cathodal stimulation decreased the reported perception of creativity.

The IRI subscales also had a significant effect on most of the considered dimensions. To be more specific, the score on the PT subscale affected how participants rated the piece to be interesting (*F*_1,34_ = 13.04, *p* = 0.001, η^2^ = 0.28), innovative (*F*_1,34_ = 37.70, *p* < 0.001, η^2^ = 0.53), and exciting (*F*_1,34_ = 31.88, *p* < 0.001, η^2^ = 0.48). The score on the FS subscale significantly affected the rating for innovation (*F*_1,34_ = 14.70, *p* = 0.001, η^2^ = 0.30) and excitement (*F*_1,34_ = 17.81, *p* < 0.001, η^2^ = 0.34). The EC subscale scores affected how interesting (*F*_1,34_ = 16.93, *p* < 0.001, η^2^ = 0.33), innovative (*F*_1,34_ = 8.84, *p* = 0.005, η^2^ = 0.21), and exciting (*F*_1,34_ = 14.75, *p* = 0.001, η^2^ = 0.30) the piece was perceived by participants, and the same was true for the PD scale: interesting (*F*_1,34_ = 41.92, *p* < 0.001, η^2^ = 0.55), innovative (*F*_1,34_ = 94.16, *p* < 0.001, η^2^ = 0.73), and exciting (*F*_1,34_ = 60.01, *p* < 0.001, η^2^ = 0.64).

Focusing on the self-report emotional response to the piece, cathodal stimulation significantly affected emotions with negative valence (*F*_1,34_ = 17.93, *p* < 0.001, η^2^ = 0.34). Cathodal stimulation decreased the intensity of negative emotions reported by participants.

All the IRI subscales other than FS affected the rating of emotions with negative valence: PT (*F*_1,34_ = 15.96, *p* < 0.001, η^2^ = 0.32), EC (*F*_1,34_ = 11.02, *p* = 0.002, η^2^ = 0.24), and PD (*F*_1,34_ = 24.41, *p* < 0.001, η^2^ = 0.42).

### Mediation Effect of Empathy

Since in the previous analyses the IRI subscales had a significant effect as covariates, we ran further analyses to explore in more detail the possible mediation effects that these subscales had on our main variables.

Using the software JAMOVI 1.2.3, we ran a GLM mediation model using as dependent variables (one for each model) those that had significant results in the previous analyses (i.e., evaluation of how innovative the piece was, evaluation of how exciting the piece was, and self-report of emotions with negative valence), the tDCS condition as independent variable, and the IRI subscales as mediators.

**FIGURE 1:**
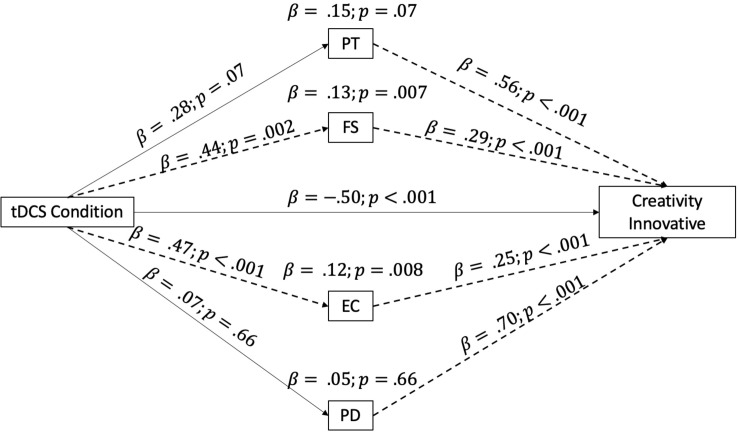
Mediation model taking into consideration the IRI subscales as mediators of the relationship between the tDCS condition and the evaluation of creativity/innovation. The arrow indicates the direction of the mediation, and the dotted lines highlight significant mediation effects. IRI subscales: PT, perspective taking; FS, fantasy; EC, empathic concern; PD, personal distress.

The first model analyzed the effect of our independent variable (tDCS condition) on the evaluation of the creativity of the music based on how innovative it was, taking into account the role of the IRI empathy subscales as mediators. The full model is reported in Figure 1.

As can be derived from the indirect effects reported in Figure 1, the FS and EC subscales appeared to be the ones who significantly mediated the effect of tDCS, by increasing the level of creative evaluation (focus on innovation).

**FIGURE 2:**
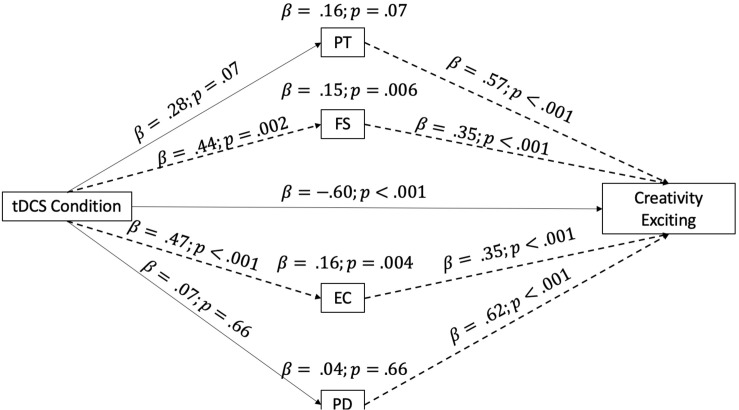
Mediation model taking into consideration the IRI subscales as mediators of the relationship between the tDCS condition and the evaluation of creativity/excitement. The arrow indicates the direction of the mediation, and the dotted lines highlight significant mediation effects. IRI subscales: PT, perspective taking; FS, fantasy; EC empathic concern; PD personal distress.

The second model analyzed the effect of our independent variable (tDCS condition) on the evaluation of the creativity of the music based on how exciting it was, considering the role of the IRI empathy subscales as moderators. The full model is reported in Figure 2. As can be seen from the indirect effects reported in Figure 2, the same two mediators (the FS and EC subscales) had a significant effect on the evaluation of creativity (focus on excitement), by increasing the level of creative evaluation.

**FIGURE 3:**
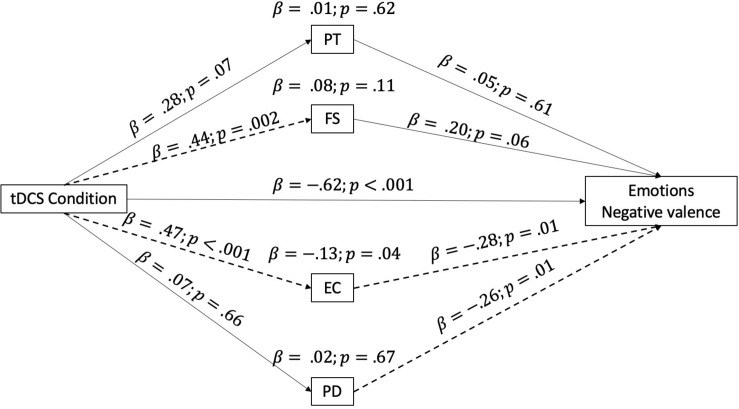
Mediation model taking into consideration the IRI subscales as mediators of the relationship between the tDCS condition and the self-report of emotions (negative valence). The arrow indicates the direction of the mediation, and the dotted lines highlight significant mediation effects. IRI subscales: PT, perspective taking; FS, fantasy; EC, empathic concern; PD, personal distress.

The last model analyzed the effect of our independent variable (tDCS condition) on the self-reported evaluation of emotions (negative valence), considering the role of the IRI empathy subscales as mediators. The full model is reported in Figure 3. In this case, the subscale EC appeared to have a significant effect, by decreasing the intensity of emotions with negative valence reported by participants.

## Discussion and Conclusion

The present study aimed at investigating the role of the auditory mirror system in influencing the evaluation of creativity as well as the emotional reactions of professional musicians while listening to a new piece of music.

Our first hypothesis focused on the effect of cathodal stimulation in reducing the perceived creativity of the new piece of music. This hypothesis was guided by the fact that evidence from literature supports the idea that the auditory MNS plays a role in musicians’ response to music (Bangert et al., 2006; Lahav et al., 2007) and also that the MNS’s role is linked to processing not only motor information but also emotional and other higher-level cognitive information, like creativity (Ramachandra et al., 2009). Moreover, empathy has been reported to influence the evaluation of creativity levels of performing arts, including music (Wöllner, 2012), and the MNS is involved in empathic responses (Gazzola et al., 2006; Baird et al., 2011; Schnell et al., 2011). For these reasons, we believed that modulating the activation of musicians’ MNS would have led to differences in their evaluation of the creativity level of a new piece of music. Our results partially confirmed this hypothesis. Participants who underwent cathodal stimulation rated the piece as less innovative and exciting when compared to participants in the sham condition. On the other hand, their evaluation of the level of interest was not significantly affected by the stimulation. Our data seem to confirm a role of the MNS in evaluating the creativity of a music piece, but the role seems to be rather specific. Both the cognitive evaluation of the creative process (the innovation of the piece) and the emotional reaction to it (excitement) appear to be influenced by the activation of the MNS. When the activation is disrupted (lowered by cathodal stimulation), the piece is perceived as less innovative and less exciting. On the other end, how interesting the piece is appears to be examined through a different circuit. We might hypothesize that this evaluation can be related to individual differences, hence not being directly affected by the modulation of the MNS. This reading is supported by research data stating that music preference is significantly influenced by a combination of the individuals’ perception of the cognitive, emotional, and cultural functions of music, together with physiological arousal and familiarity (Schäfer and Sedlmeier, 2010). Further research might include evaluation of these variables into a tDCS design similar to the one presented in this study to better assess their specific role. A possible reading of the non-significant results concerning how interesting the piece was is related to the type of assessment used in this study. It has been reported that interest is directly linked to participants’ level of attention (Peters et al., 2005; Li et al., 2020), something that we did not control for in our study. This aspect should be taken into consideration in future studies.

The above-mentioned results regarding the effect of brain stimulation on the evaluation of how exciting a music piece is stresses a conceptual link with our second hypothesis, which focuses on the effect of tDCS on participants’ reported emotions after listening to the music. This hypothesis was formulated based on the evidence (Warren et al., 2006; Banissy et al., 2010) that the auditory MNS plays a significant role in responding to auditory stimuli with emotional valance. Our hypothesis was partially confirmed. To be more precise, it was confirmed only for emotion with a negative valence (after cathodal stimulation, people reported less emotion with a negative valence) but not for emotions with positive valence. There are two possible explanations for this result. The first one refers to the specific music we were using for our study. Even if the movement that we used was not written with a specific emotional tone, it has a slow tempo, and it is mainly written in the tonality of D minor. Minor keys and lower tempos tend to be associated with more negative emotions like sadness (Webster and Weir, 2005), so the effect of neuromodulation might have been more pronounced for these specific emotions. Also, fMRI data suggest that familiarity seems to play an important role in making the listeners emotionally engaged with music (Pereira et al., 2011), and our piece was unfamiliar to all our participants. Future studies should add a familiar piece as a comparison, to understand if increased familiarity might lead to a different result.

Our last hypothesis was linked to the possible mediating effect of individual empathy levels, inspired by the fact that studies show positive correlations between the activation of the MNS and empathy scores from the IRI (Baird et al., 2011). Results from our mediation models supported this hypothesis and highlighted the specific role of different empathy traits. Two IRI subscales appeared to mediate the effect of brain stimulation by increasing the evaluation of creativity, even after cathodal stimulation: EC (tendency to experience feelings of sympathy and compassion for unfortunate others) and FS (tendency to imaginatively transpose oneself into fictional situations). Previous studies reported similar findings when focusing only on the role of trait empathy in music appreciation. For example, Garrido and Schubert (2011) reported a significant correlation between EC scores and liking sad music. In another study (Vuoskoski et al., 2011), a similar positive correlation between the same subscales that were reported as significant moderators in our study (EC and FS) and music appreciation was found. A similar result emerged when focusing on the mediating effect of trait empathy on perceived emotions, with a significant moderating role of EC emerging but only for negative-valence emotions. Altogether, results from the mediation models seem to imply that specific empathic traits might help the listener enjoy music that is perceived as sad by increasing daydreaming about a fictional world dominated by the emotional valence elicited by the music and experience compassion inspired by the feelings suggested by the music. Future studies should test this reading by adding a measure of visual imagery experiences to the research design and testing the same design with a music written in a major key and with a faster tempo.

### Conclusion

The present study presents some interesting, if preliminary, data on the role of the auditory MNS in mediating the cognitive and emotional response to music of professional musicians. In particular, we were able to highlight a role of the auditory MNS in evaluating specific aspects of musical creativity (innovation and excitement) and in influencing, at least partially, the emotional response to the same music. Moreover, we were able to highlight the specific mediating role of trait empathy. From a theoretical standpoint, our results offer more evidence to better clarify the role of the auditory MNS in evaluating music, as well as highlighting some more insights into the specific role of the subcomponents of empathy in mediating the cognitive and affective responses of the MNS—something that, as highlighted in our literature review, is still in need of additional clarification (Baird et al., 2011).

From an applied standpoint, the results offer some interesting implications for the use of music to promote creativity as well as social skill in different educational settings. The relationship between creativity and empathy within the response to music could be used to support specific programs aimed at working with youths with autism spectrum disorders (Forti et al., 2020) but could also be used to inform assessment in music composition (Deutsch, 2016).

These results are promising and worth being further explored by future studies. Yet some additional limitations should be highlighted. Our study did not explore the effect of anodal stimulation and focused only on the evaluation of one piece of music, which was perceived by participants as sad because of the specific tempo and key. Future studies should explore the effects of anodal stimulation, as well as add information about the effects of the auditory MNS in mediating the creative evaluation and emotional response to music that is perceived as happy. Moreover, we worked with a sample of professional but young musicians. Future studies should involve older musicians to test if expertise might play an additional moderation role. Finally, even if we achieved a good effect size (as can been derived by the η^2^ values), we have been working with a relatively small sample. Future studies should aim at collecting data from a larger sample.

## Data Availability Statement

The raw data supporting the conclusions of this article will be made available by the authors, without undue reservation.

## Ethics Statement

The studies involving human participants were reviewed and approved by Champlain College IRB. The patients/participants provided their written informed consent to participate in this study.

## Author Contributions

BC designed the study, collected data, run analyses, and wrote the manuscript. RA designed the study and wrote the manuscript. SB analyzed the data and wrote the manuscript. FB designed the methodology and wrote the manuscript. AA designed the study and supervised the study. All authors contributed to the article and approved the submitted version.

## Conflict of Interest

The authors declare that the research was conducted in the absence of any commercial or financial relationships that could be construed as a potential conflict of interest.
